# Investigating the effectiveness of commercially available mouthwash on SARS-CoV-2 in vivo using viable virus titre as the primary outcome. A randomised controlled trial

**DOI:** 10.1099/acmi.0.000722.v3

**Published:** 2024-07-08

**Authors:** D.W. Seymour, G. Forshaw, M. Porteous, D. Mawer, F. Wiggins, A. Mitchell, C. Hewitt, T. Beetar-King, K.A. Davies, D. Jackson, M.J. Hannah, M. Pitcher, U. Arnold, R. Strachan, M.J. Killip, P.J. Nixon

**Affiliations:** 1 York and Scarborough Teaching Hospitals NHS Foundation Trust, Wigginton Road, York, YO31 8HE, UK; 2 York Trials Unit, Department of Health Sciences, ARRC Building, University of York, Heslington, York, YO10 5DD, UK; 3 High Containment Microbiology, United Kingdom Health Security Agency, 61 Colindale Avenue, Colindale, London, NW9 5EQ, UK

**Keywords:** mouthwash, saliva, SARS-CoV-2

## Abstract

This multi-arm, parallel group, single-blinded randomised controlled trial aimed to assess three commercially available mouthwashes effectiveness against severe acute respiratory syndrome coronavirus 2 (SARS-CoV-2). This manuscript has been written in accordance with the CONSORT statement.

**Methods.** Eligible participants were SARS-CoV-2 positive with a positive test in the last 72 h. All participants had mild to moderate symptoms and could provide five saliva samples over a 60 min period. Participants delivered a baseline saliva sample and then used a mouthwash as per manufacturer’s instructions. They provided further saliva samples at minute 1, 10, 30 and 60. Participants were randomised to one of four groups; OraWize+, Total Care Listerine, Cool Mint Listerine and water (control). The lab-based research team were blind to the intervention. The research question was: can SARS-CoV-2 be rendered inactive in saliva by using a mouthwash and how long does this effect last? The primary outcome was the amount of viable infectious SARS-CoV-2 virus in the sample, compared to the baseline sample. The secondary outcome measure was the amount of genetic material from the SARS-CoV-2 virus in the sample, measured via PCR testing.

**Results.** In total 100 participants were recruited (25 per group). Eight participants did not receive the allocated intervention and did not have saliva samples collected. There were no adverse events. In total 42 of the 92 participants had viable virus which could be cultured at baseline. Statistical analysis of the primary outcome was not advised due to the reduced level of viable virus at baseline and the positive skewness present in the distribution of log10(titre) data. Observational data of the primary outcome measure is presented.

Analysis of the secondary outcome PCR measure showed that there was strong evidence for a decrease in SARS-CoV-2 RNA levels compared to water for all mouthwashes after 1 min, OraWize+ −0.49 (−0.92, –0.05), *p*-value 0.029, Cool Mint Listerine −0.81 (−1.25, –0.38), *p*-value<0.001, Total Care Listerine −1.05 (−1.48, –0.62), *p*-value<0.001. For the remaining timepoints there was generally no evidence of virus level reduction compared to water although there is weak evidence for a decrease at ten minutes using Total Care Listerine −0.44 (−0.88, 0.01), *p*-value 0.053.

**Conclusion.** The three mouthwashes included in this trial observationally demonstrated a reduction in virus titre level 1 min after use, with virus levels normalising up to 60 min compared to the control. Although an interesting observation, this result could not be statistically analysed. Using the secondary outcome PCR measure all three included mouthwashes reduced virus levels compared to water at 1 min and these results were statistically significant. Clinically this result does not support the use of the included mouthwashes to reduce SARS-CoV-2 levels in saliva.

## Data Summary

We have supplied extensive supportive anonymised data in Tables 1–7 and Figures 1 and 2. Patient sensitive data has not been supplied due to ethical considerations.

## Introduction

### Background

Severe acute respiratory syndrome coronavirus 2 (SARS-CoV-2) emerged in late 2019. SARS-CoV-2 is the virus responsible for causing the COVID-19 pandemic [1]. Symptoms of COVID-19 range from asymptomatic infection or mild, transient symptoms to severe respiratory failure [2].

SARS-CoV-2 has been shown to be present in saliva in significant quantities [3,4]. High levels of virus in the saliva increases the risk of cross infection with medical or dental procedures involving the mouth. The risk is thought to increase when performing aerosol generating procedures [5]. Pre-procedural mouthwashes have proven efficacy reducing bacteria in aerosols [6,8].

An initial *in vitro* study by the authors tested various commercially available mouthwashes against SARS-CoV-2. This demonstrated that numerous commercially available mouthwashes inactivate SARS-CoV-2 [9]. Similar studies have confirmed this in a laboratory setting [10,12]. This clinical *in vivo* study included the three mouthwashes that were confirmed effective in the *in vitro* study and tested their effectiveness against water using a multi-arm, parallel group, single-blinded randomised controlled trial. Mouthwashes that were not commercially available in the UK were not analysed, for example, povidone-iodine containing mouthwashes.

### Objectives

The primary objective was to assess whether SARS-CoV-2 could be rendered inactive in saliva by using a mouthwash and estimate how long such an effect may last.

## Methods

### Trial design

The trial was a multi-arm, parallel group, single-blinded, randomised controlled trial.

### Participants

To be eligible to be included in the trial, participants were initially required to have had a positive SARS-CoV-2 PCR test in the 72 h prior to randomisation. In addition, participants needed to display mild to moderate symptoms of SARS-CoV-2. This inclusion criterion was later amended to accept participants with positive SARS-CoV-2 lateral flow tests, and a later amendment required that participants confirm positivity with a lateral flow before sampling on the day.

Those too unwell to participate or provide saliva samples were excluded. Participants with an allergy to any of the ingredients in the study mouthwashes and pregnant women were also excluded.

### Study settings

Participants were recruited and saliva samples were collected at York Hospital, England. Samples were collected at a drive-through testing site and on the hospital wards by members of the hospital research team. Laboratory analysis was conducted at the United Kingdom Health Security Agency (UKHSA; Colindale, London).

### Registration

The trial was registered with ISRCTN registry in June 2021 (ISRCTN 16269648). The study protocol can be found on the registry

### Intervention

An initial *in vitro* study by the authors demonstrated numerous commercially available mouthwashes inactivate SARS-CoV-2 [9]. The most effective mouthwashes were included for use in this study; OraWize+, Total Care Listerine and Cool Mint Listerine. The control was water. The potential active ingredients contained in the mouthwashes are hypochlorous acid, alcohol, eucalyptol, thymol, menthol, sodium fluoride and zinc chloride.

Following consent and randomisation, patients provided an initial baseline saliva sample by spitting 5 ml of saliva into a 60 ml saliva pot. The participant then used their allocated 10 ml of mouthwash or water for 60 s and spat the excess in a disposable bowl. Further 5 ml saliva samples were then provided in the same way that the baseline sample was collected at 1 min, 10 min, 30 min and 60 min. Samples were kept on ice and once sampling was complete the samples were immediately frozen at minus eighty degrees Celsius. All sample pots were labelled with the patient’s unique trial ID.

Samples were transferred frozen to the United Kingdom Health Security Agency High Containment Microbiology lab in London for lab analysis. Laboratory staff were blinded to study allocation.

### Outcomes

The primary outcome was the amount of viable infectious SARS-CoV-2 virus in the sample, measured via virological titration assays and reported as the logarithm of the focus forming units per millilitre (log_10_FFU ml^−1^). In this study our objective was to quantitatively assess the effect of commercial mouthwashes on viable SARS-CoV-2 using virus culture.

A secondary outcome measure was the amount of genetic material from the SARS-CoV-2 virus in the sample, measured via PCR testing and reported as the logarithm (base 10) of the number of RNA copies per millilitre. Each sample was processed twice to improve accuracy in measuring the outcomes, with the final value for each outcome being the mean of the two samples.

## Laboratory analysis

All handling and processing of non-inactivated samples was carried out within class III microbiological safety cabinets in a Containment Level three facility.

Levels of viable SARS-CoV-2 in saliva samples were assessed quantitatively by determining the infectious virus titre by immunofocus assay. In brief, duplicate half-log dilution series of each sample were prepared and 100 µl of each dilution inoculated onto 24-well plates of VeroE6-ACE2-TMPRSS2 cells (provided by the NIBSC Research Reagent Repository, UK. With thanks to Professor Arvind Patel, MRC-University of Glasgow Centre for Virus Research, University of Glasgow). A plate comprising SARS-CoV-2 of a known virus titre was included in every infection batch as a positive infection and immunostaining control. Virus was adsorbed at ambient temperature for 1 h, followed by addition of a 1 % avicel overlay. Plates were incubated at 37°C/5 % CO_2_. Plates were fixed with formaldehyde after 24 h. Fixed plates were treated with 1 % hydrogen peroxide in methanol, to permeabilise cells and to block endogenous peroxidases. Plates were then stained with rabbit anti-SARS-CoV-2 nucleoprotein antibody (Sino Biological) followed by a goat anti-rabbit IgG-peroxidase conjugate (Sigma-Aldrich), both in 5 % skimmed milk powder/PBS-Tween, with thorough washing of monolayers with PBS-Tween after each step. TrueBlue peroxidase substrate (KPL) was added to monolayers to visualise SARS-CoV-2-specific staining. Plates were imaged using an Immunospot S6 analyser (CTL), and virus foci manually counted from images to determine virus titre for each sample duplicate.

Levels of SARS-CoV-2 nucleic acid in samples was determined as follows. Duplicate 70 µl volumes were taken from each sample and inactivated in Buffer AVL (QIAGEN) for 10 min, followed by addition of absolute ethanol (at 1 vol sample: 4 volumes Buffer AVL: 4 volumes ethanol) before removal of inactivated samples from containment. Buffer AVL was spiked with MS2 bacteriophage as an internal extraction and PCR control. RNA was extracted from inactivated samples using a Viral RNA Mini Kit (QIAGEN), according to manufacturer’s instructions. RNA extracts were tested for presence of the SARS-CoV-2 E gene and MS2 RNA by multiplexed real-time reverse-transcriptase PCR [13] on an Applied Biosystems 7500 Fast platform. Alongside sample RNA extracts, a dilution series of E RNA transcript with a known number of copies per microlitre was run for creation of a standard curve and conversion of Ct values into RNA copies.

### Sample size

Sample size calculation was difficult given the lack of available data and the nature of the COVID-19 pandemic at the start of the trial. A power calculation gave a sample size of 23 participants per group, with 90 % power to show a difference of one standard deviation in the viral load and a 35 % difference (0–35 %) clearance rate.

### Randomisation

Participants were randomly assigned to each group via non-stratified block randomisation using block sizes of four. The tool used was www.randomization.com.

### Allocation concealment

On recruitment to the study each participant was assigned a unique Trial ID. The member of the research team taking consent assigned the next sequential number and then documented in the Trial Master File the Trial ID and corresponding NHS number of the participant. This allowed reconciliation of trial results at the end of the trial. The mouthwash the participant was assigned to was in a numbered container to allow allocation concealment. The research team member collecting the sample was different to the team member consenting the patient. The sequence was randomly generated, and the consenting team member allocated the participant to the next trial number sequentially.

### Randomisation: implementation

Sequence generation was completed by a study co-ordinator. Allocation concealment was completed by a different study co-ordinator. The mouthwash interventions were kept in identical, unbranded pre-prepared 60 ml clear saliva containers.

The trial was discussed with participants by the research nurse who also gained participant consent to enrol in the study. The saliva samples were collected by various members of the team including research nurses, research health care providers and study co-ordinators. The mouthwash and instructions were simply given to the participants by the team; at no stage did the team member (or the participant) know which group the participant had been assigned to.

### Blinding

All team members who provided the mouthwash and collected the saliva samples were blinded to the intervention group. Participants were blinded to the mouthwash by using unbranded containers. However, the intervention mouthwashes did contain a flavour.

The UKHSA laboratory team analysing the saliva samples were all blinded to the intervention group. They only had access to the trial ID number and the saliva samples. The University of York academic team providing statistical support and York Hospital team were unblinded at the statistical analysis stage.

### Statistical analysis

All analyses were carried out with Stata Version 17.0. Statistical hypothesis tests were two-sided and used a 5 % significance level. Baseline and outcome data are summarised descriptively by randomised group. Continuous data are summarised using means, standard deviations, median, the IQR, minimum and maximum. Categorical data is summarised using counts and percentages.

The threshold level of detection for the primary outcome was <10 FFU ml^−1^. For the purposes of the analyses, samples below threshold level were assumed to have a value of 5 FFU ml^−1^. It was intended to analyse both the primary and secondary outcomes using a repeated-measures, mixed effects linear regression model adjusting for the baseline value of the outcome. Model assumptions of homoscedasticity and normality of residuals were assessed graphically. Due to the skewed distribution of the data, it was not possible to analyse the primary outcome in this manner. However, the assumptions of the planned analysis of the secondary outcome were met and the repeated-measures, mixed-effects model analysis was carried out. An unstructured correlation structure was found to minimise the Akaike Information Criterion and lead to the best model fit. Estimates of the pairwise difference between each of the mouthwashes of interest compared to water are presented alongside 95 % confidence intervals and *p*-values. A sensitivity analysis was carried out excluding participants who were PCR negative at baseline from the secondary outcome analysis.

## Results

### Recruitment

Trial recruitment took place between 28 June 2021 to 16 June 2022. In total, 100 participants were recruited and randomised to the study (Fig. 1).

### Participant flow

**Fig. 1.**
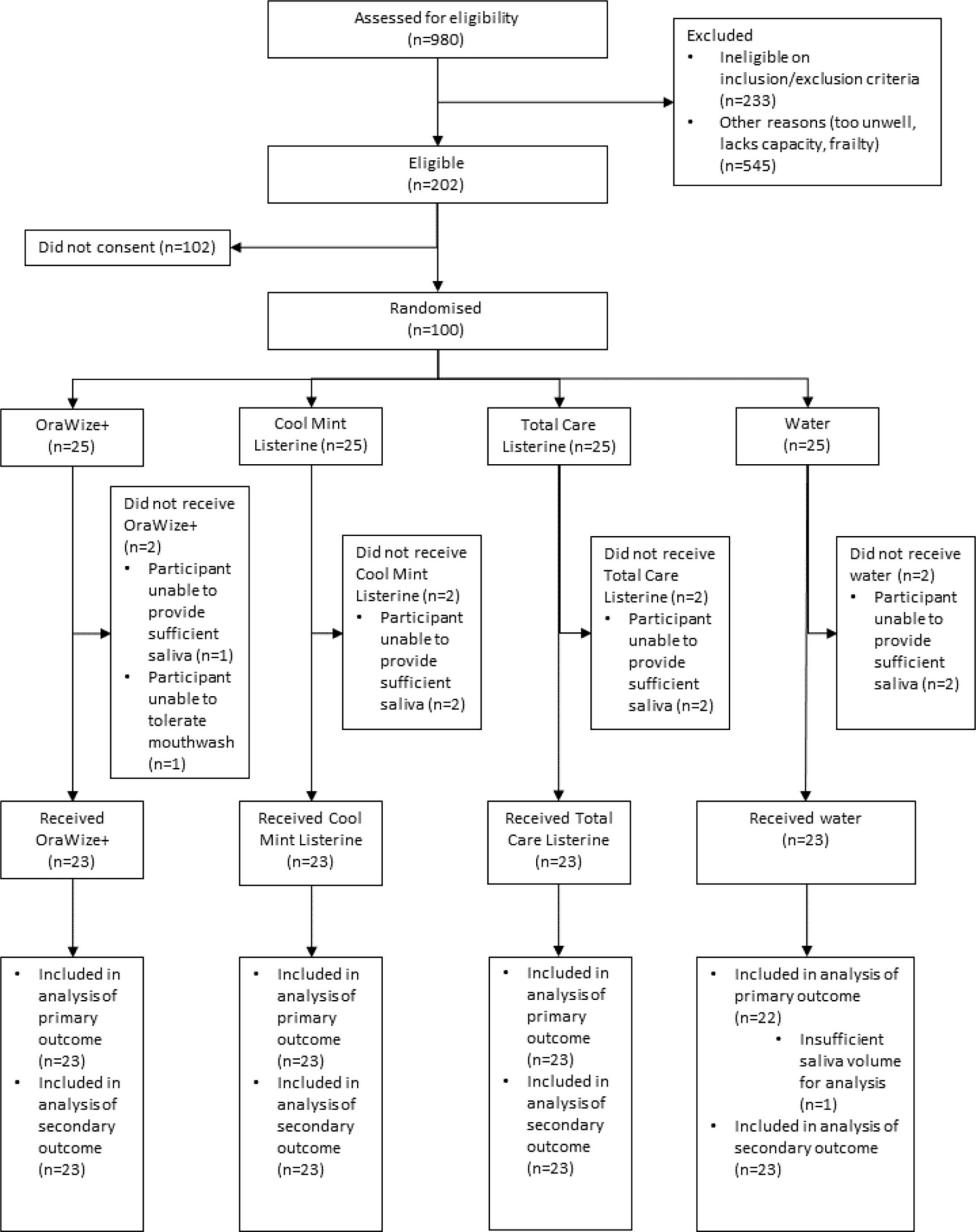
Flow diagram demonstrating the progression of the trial.

### Baseline data

Table 1 gives information on the type of participant and test results overall and by group. In total, 44 (47.8 %) participants were hospitalised patients, while 48 (52.2 %) were members of the public. The average of the logarithm of the number of focus forming units per millilitre was 1.6 FFU ml^−1^ (SD 1.4). The average of the logarithm (base 10) of the number of RNA copies was 6.2 per millilitre (SD 1.4).

**Table 1.** Information on participant recruitment pathway and baseline test results, presented overall and by group

	OraWize+(*n*=25)	Cool Mint Listerine(*n*=25)	Total Care Listerine(*n*=25)	Water(*n*=25)	Total(*n*=100)
Recruitment pathway, n (%)					
*Number with data*	*23(92.0)*	*23(92.0)*	*23(92.0)*	*23(92.0)*	*92(92.0)*
Hospitalised patient	10 (43.5)	11 (47.8)	11 (47.8)	12 (52.2)	44 (47.8)
Member of public	13 (56.5)	12 (52.2)	12 (52.2)	11 (47.8)	48 (52.2)
Raw sample CT					
*Number with data (%)*	*23(92.0)*	*23(92.0)*	*23(92.0)*	*23(92.0)*	*92(92.0)*
Mean (SD)	26.9 (4.2)	25.1 (5.6)	26.4 (3.8)	27.4 (5.3)	26.5 (4.8)
Median (IQR)	26.0 (24.8, 29.8)	25.1 (21.8, 28.2)	27.2 (22.8, 29.0)	27.5 (22.1, 33.5)	26.0 (23.2, 29.6)
Min, Max	19.2, 34.5	13.6, 34.5	20.7, 34.5	18.1, 34.5	13.6, 34.5
Log_10_(Average number of RNA copies ml^−1^)					
*Number with data (%)*	*23(92.0)*	23 *(92.0)*	*23(92.0)*	*23(92.0)*	*92(92.0)*
Mean (SD)	6.1 (1.2)	6.6 (1.7)	6.2 (1.1)	5.9 (1.5)	6.2 (1.4)
Median (IQR)	6.4 (5.4, 6.6)	6.6 (5.7, 7.5)	6.1 (5.3, 7.5)	5.8 (4.3, 7.5)	6.3 (5.3, 7.1)
Min, Max	3.8, 8.2	3.8, 9.8	3.8, 7.9	3.8, 8.5	3.8, 9.8
COVID-19 baseline PCR test status, n (%)					
*Number with data (%)*	*23(92.0)*	*23(92.0)*	*23(92.0)*	*23(92.0)*	*23(92.0)*
Positive	21 (91.3)	22 (95.7)	22 (95.7)	20 (87.0)	85 (92.4)
Negative/undetermined	2 (8.7)	1 (4.3)	1 (4.3)	3 (13.0)	7 (7.6)
Log_10_(FFU ml^−1^)					
*Number with data (%)*	*23(92.0)*	*23(92.0)*	*23(92.0)*	*22(88.0)*	*91(91.0)*
Mean (SD)	1.4 (1.5)	1.8 (1.6)	1.7 (1.6)	1.5 (1.1)	1.6 (1.4)
Median (IQR)	0.7 (0.7, 1.7)	0.9 (0.7, 2.9)	0.9 (0.7, 2.2)	0.8 (0.7, 2.0)	0.7 (0.7, 2.0)
Min, Max	0.7, 7.3	0.7, 6.5	0.7, 6.6	0.7, 5.2	0.7, 7.3
Titre results below detectable threshold of 10 FFU ml^−1^, n (%)					
*Number with data (%)*	*23(92.0)*	*23(92.0)*	*23(92.0)*	*22(88.0)*	*91(91.0)*
Yes	16 (69.6)	11 (47.8)	11 (47.8)	11 (50.0)	49 (53.9)
No	7 (30.4)	12 (52.2)	12 (52.2)	11 (50.0)	42 (46.2)

### Numbers analysed

Eight participants did not receive the intervention and as a result did not have saliva samples collected (OraWize+ 2; Cool Mint Listerine 2; Total Care Listerine 2; water 2). Seven participants were unable to provide sufficient saliva and one participant could not tolerate the allocated mouthwash.

### Outcome and estimation

The raw data showing the primary outcome titre values are presented in Table 2. The raw data showing the secondary outcome PCR values are shown in Table 3.

**Table 2.** Logarithm (base 10) of the focus forming units per millilitre presented by group

	OraWize+(*n*=25)	Cool Mint Listerine(*n*=25)	Total Care Listerine(*n*=25)	Water(*n*=25)
**1 min**				
*Number with data (%)*	*23(92.0)*	*23(92.0)*	*23(92.0)*	*22(88.0)*
Mean (SD)	1.2 (0.9)	1.1 (0.8)	0.9 (0.8)	1.4 (1.2)
Median (IQR)	0.7 (0.7, 1.4)	0.7 (0.7, 1.0)	0.7 (0.7, 0.7)	0.8 (0.7, 1.9)
Min, Max	0.7, 4.1	0.7, 4.2	0.7, 4.5	0.7, 4.9
**10** **min**				
*Number with data (%)*	*23(92.0)*	*23(92.0)*	*23(92.0)*	22 *(88.0)*
Mean (SD)	1.2 (1.1)	1.7 (1.7)	1.4 (1.2)	1.5 (1.0)
Median (IQR)	0.7 (0.7, 1.0)	0.9 (0.7, 3.0)	0.7 (0.7, 2.0)	1.4 (0.7, 2.0)
Min, Max	0.7, 4.9	0.7, 7.9	0.7, 4.6	0.7, 4.6
**30** **min**				
*Number with data (%)*	*23(92.0)*	*23(92.0)*	*23(92.0)*	*22(88.0)*
Mean (SD)	1.3 (1.1)	1.6 (1.5)	1.5 (1.3)	1.5 (1.1)
Median (IQR)	0.7 (0.7, 1.5)	0.7 (0.7, 2.3)	0.7 (0.7, 2.3)	0.8 (0.7, 2.3)
Min, Max	0.7, 4.7	0.7, 6.4	0.7, 5.3	0.7, 5.0
**60** **min**				
*Number with data (%)*	*23(92.0)*	*22(88.0)*	*23(92.0)*	*22(88.0)*
Mean (SD)	1.3 (1.3)	1.6 (1.5)	1.6 (1.4)	1.6 (1.3)
Median (IQR)	0.7 (0.7, 1.4)	0.7 (0.7, 2.0)	0.7 (0.7, 2.4)	0.8 (0.7, 2.0)
Min, Max	0.7, 6.4	0.7, 5.5	0.7, 5.4	0.7 4.7

**Table 3.** Logarithm (base 10) of the RNA copies per millilitre presented by group

	OraWize+(*n*=25)	Cool Mint Listerine(*n*=25)	Total Care Listerine(*n*=25)	Water(*n*=25)
**1** **min**				
*Number with data (%)*	*23(92.0)*	*23(92.0)*	*23(92.0)*	*23(92.0)*
Mean (SD)	5.8 (1.4)	5.8 (1.4)	5.3 (1.1)	6.1 (1.6)
Median (IQR)	5.9 (4.5, 6.7)	5.6 (4.8, 6.7)	5.0 (4.5, 5.7)	6.1 (4.8, 7.7)
Min, Max	3.8, 8.2	3.8, 9.2	3.8, 8.5	3.8, 8.6
**10** **min**				
*Number with data (%)*	*23(92.0)*	*23(92.0)*	*23(92.0)*	*23(92.0)*
Mean (SD)	6.3 (1.2)	6.5 (1.5)	6.0 (1.2)	6.2 (1.4)
Median (IQR)	6.5 (5.2, 7.1)	6.4 (5.6, 7.5)	6.1 (5.1, 6.6)	6.5 (4.6, 7.2)
Min, Max	3.8, 8.9	3.8, 9.6	3.8, 8.1	3.8, 8.6
**30** **min**				
*Number with data (%)*	*23(92.0)*	*23(92.0)*	*23(92.0)*	*23(92.0)*
Mean (SD)	6.5 (1.4)	6.6 (1.5)	6.3 (1.1)	6.1 (1.6)
Median (IQR)	6.8 (5.6, 7.8)	6.7 (5.7, 7.8)	6.7 (5.4, 7.3)	6.1 (4.9, 7.6)
Min, Max	3.8. 9.0	3.8, 9.7	4.0, 8.3	3.8, 9.0
**60** **min**				
*Number with data (%)*	*23(92.0)*	22 *(88.0)*	*23(92.0)*	*23(92.0)*
Mean (SD)	6.3 (1.2)	6.7 (1.4)	6.3 (0.9)	6.3 (1.6)
Median (IQR)	6.6 (5.6, 7.1)	6.8 (5.9, 7.6)	6.5 (5.4, 7.1)	6.4 (5.0, 7.7)
Min, Max	3.8, 8.8	3.8, 9.6	4.4, 7.5	3.8, 8.9

**Fig. 2.**
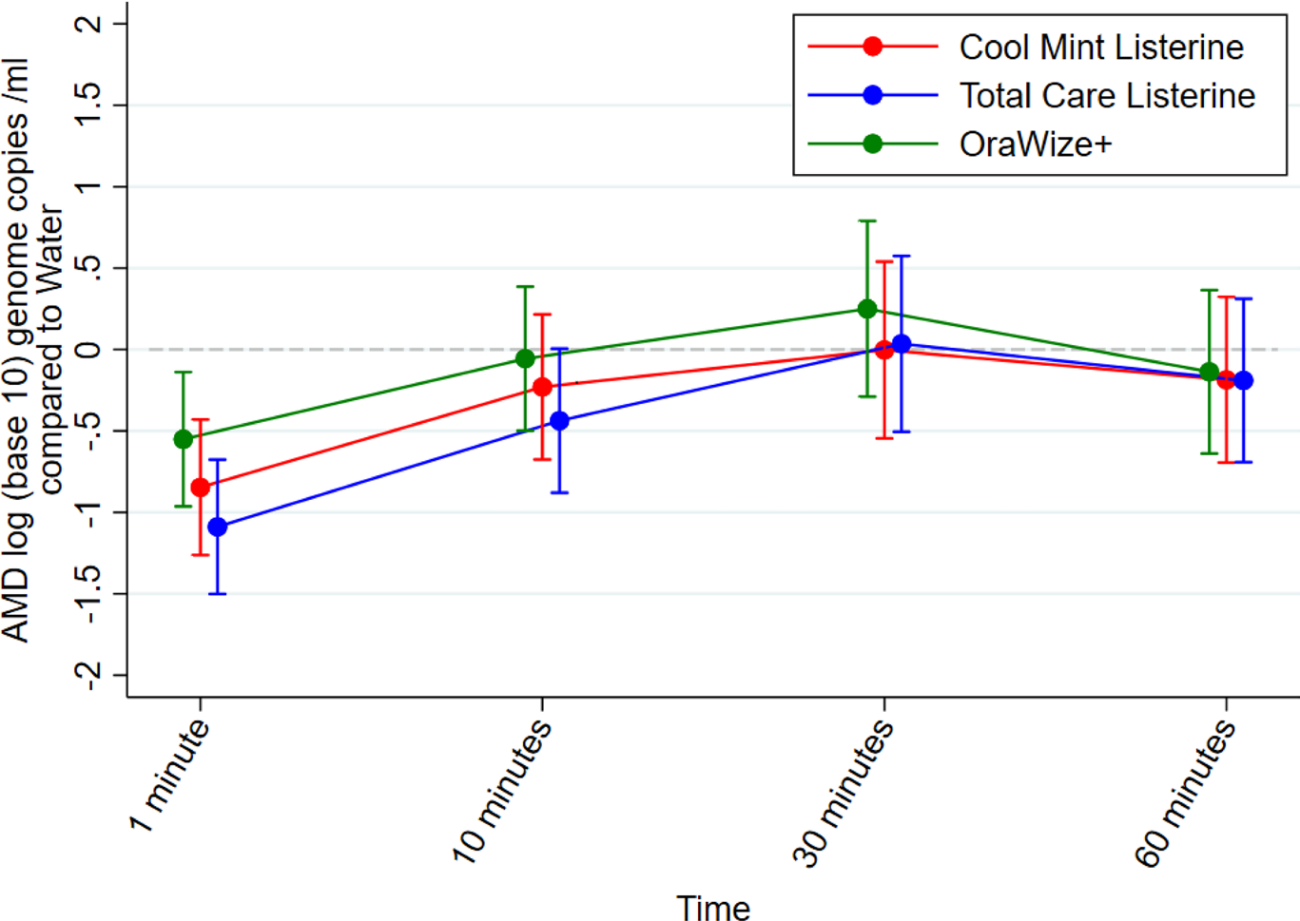
Graphic results of the analysis of the logarithm (base 10) of the RNA copies per millilitre.

Table 4 and Fig. 2 show the results of the analysis on the secondary PCR outcome measure. Given the skewed distribution of the data it was not possible to statistically analyse the primary outcome measure. There was strong evidence for a decrease in SARS-CoV-2 levels compared to water for all mouthwashes after 1 min, OraWize+ −0.49 (-0.92,–0.05), *p*-value 0.029, Cool Mint Listerine −0.81 (-1.25,–0.38), *p*-value<0.001, Total Care Listerine −1.05 (-1.48,–0.62), *p*-value<0.001. For the remaining timepoints there was generally no evidence of virus level reduction compared to water although there is weak evidence for a decrease at ten minutes using Total Care Listerine −0.44 (-0.88, 0.01), *p*-value 0.053. Overall Total Care Listerine had the biggest effect compared to water over 60 min. No mouthwash provided a substantive effect. Table 5 shows that this result is similar when the seven PCR negative participants are removed from the analysis.

**Table 4.** Results of the analysis of the logarithm (base 10) of the RNA copies per millilitre

	OraWize+ vs water	Cool Mint Listerine vs water	Total Care Listerine vs water
**1** **min**			
AMD (95 % CI)	−0.55 (−0.96, –0.14)	−0.85 (−1.26, –0.43)	−1.09 (−1.50, –0.68)
*p*-value	0.009	<0.001	<0.001
**10** **min**			
AMD (95 % CI)	−0.06 (−0.50, 0.39)	−0.23 (−0.68, 0.22)	−0.44 (−0.88, 0.01)
*p*-value	0.803	0.312	0.053
**30** **min**			
AMD (95 % CI)	0.25 (−0.29, 0.79)	0.00 (−0.55, 0.54)	0.03 (−0.50, 0.57)
*p*-value	0.364	0.991	0.899
**60** **min**			
AMD (95 % CI)	−0.14 (−0.64, 0.36)	−0.19 (−0.69, 0.32)	−0.19 (−0.69, 0.31)
*p*-value	0.592	0.474	0.458

**Table 5.** Results of the analysis of the logarithm (base 10) of the RNA copies per millilitre, (participants with positive PCR test results only)

	OraWize+ vs water	Cool Mint Listerine vs water	Total Care Listerine vs water
**1** **min**			
AMD (95 % CI)	−0.49 (−0.92, –0.05)	−0.81 (−1.25, –0.38)	−1.05 (−1.48, –0.62)
*p*-value	0.029	<0.001	<0.001
**10** **min**			
AMD (95 % CI)	−0.05 (−0.50, 0.40)	−0.16 (−0.60, 0.29)	−0.36 (−0.80, 0.09)
*p*-value	0.830	0.489	0.114
**30** **min**			
AMD (95 % CI)	0.32 (−0.21, 0.86)	0.07 (−0.46, 0.60)	−0.05 (−0.58, 0.48)
*p*-value	0.236	0.791	0.860
**60** **min**			
AMD (95 % CI)	−0.14 (−0.61, 0.33)	−0.08 (−0.55, 0.39)	−0.19 (−0.65, 0.28)
*p*-value	0.561	0.744	0.434

To the best knowledge of the study team all participants recruited were SARS-CoV-2 positive. Samples tested at the UKHSA showed that seven participants provided PCR negative saliva samples. Tables6  7 give raw data for titre and PCR data excluding the PCR negative samples. Table 5 shows the results of the analysis on the secondary PCR outcome measure excluding the PCR negative samples.

**Table 6.** Logarithm (base 10) of the focus forming units per millilitre presented by group (participants with positive PCR test results only)

	OraWize+(*n*=21)	Cool Mint Listerine(*n*=22)	Total Care Listerine(*n*=22)	Water(*n*=20)
**1** **min**				
*Number with data (%)*	*21* (100)	*22* (100)	*22* (100)	*20* (100)
Mean (SD)	1.2 (1.0)	1.1 (0.8)	0.9 (0.8)	1.5 (1.2)
Median (IQR)	0.7 (0.7, 1.4)	0.7 (0.7, 1.0)	0.7 (0.7, 0.7)	0.9 (0.7, 2.0)
Min, Max	0.7, 4.1	0.7, 4.2	0.7, 4.5	0.7, 4.9
**10** **min**				
*Number with data (%)*	*21* (100)	*22* (100)	*22* (100)	*20* (100)
Mean (SD)	1.2 (1.1)	1.7 (1.7)	1.4 (1.2)	1.6 (1.0)
Median (IQR)	0.7 (0.7, 1.0)	0.9 (0.7, 3.0)	0.7 (0.7, 2.0)	1.5 (0.7, 2.1)
Min, Max	0.7, 4.9	0.7, 7.9	0.7, 4.6	0.7, 4.6
**30** **min**				
*Number with data (%)*	*21* (100)	*22* (100)	*22* (100)	*20* (100)
Mean (SD)	1.3 (1.1)	1.6 (1.5)	1.5 (1.3)	1.6 (1.1)
Median (IQR)	0.7 (0.7, 1.5)	0.7 (0.7, 2.2)	0.7 (0.7, 2.3)	1.2 (0.7, 2.3)
Min, Max	0.7, 4.7	0.7, 6.4	0.7, 5.3	0.7, 5.0
**60** **min**				
*Number with data (%)*	*21* (100)	*21* (95.5)	*22* (100)	*20* (100)
Mean (SD)	1.3 (1.3)	1.6 (1.5)	1.7 (1.4)	1.7 (1.4)
Median (IQR)	0.7 (0.7, 1.4)	0.7 (0.7, 2.0)	0.9 (0.7, 2.4)	0.9 (0.7, 2.1)
Min, Max	0.7, 6.4	0.7, 5.5	0.7, 5.4	0.7, 4.7

**Table 7.** Logarithm (base 10) of the RNA copies per millilitre presented by group (participants with positive PCR test results only)

	OraWize+(*n*=21)	Cool Mint Listerine(*n*=22)	Total Care Listerine(*n*=22)	Water(*n*=20)
**1** **min**				
*Number with data (%)*	*21* (100)	*22* (100)	*22* (100)	*20* (100)
Mean (SD)	6.0 (1.3)	5.9 (1.4)	5.3 (1.1)	6.3 (1.6)
Median (IQR)	6.0 (5.4, 6.7)	5.8 (4.9, 6.7)	5.2 (4.7, 5.7)	6.2 (4.9, 7.7)
Min, Max	3.8, 8.2	3.8, 9.2	3.8, 8.5	3.8, 8.6
**10** **min**				
*Number with data (%)*	*21* (100)	*22* (100)	*22* (100)	*20* (100)
Mean (SD)	6.4 (1.2)	6.6 (1.4)	6.1 (1.1)	6.4 (1.4)
Median (IQR)	6.5 (5.3, 7.1)	6.5 (5.7, 7.5)	6.2 (5.1, 6.6)	6.5 (5.1, 7.4)
Min, Max	4.3, 8.9	4.1, 9.6	4.1, 8.1	3.8, 8.6
**30** **min**				
*Number with data (%)*	*21* (100)	*22* (100)	*22* (100)	*20* (100)
Mean (SD)	6.7 (1.3)	6.7 (1.5)	6.3 (1.0)	6.3 (1.6)
Median (IQR)	6.8 (5.8, 7.8)	6.8 (5.8, 7.8)	6.6 (5.4, 7.1)	6.5 (5.2, 7.6)
Min, Max	3.9, 9.0	3.8, 9.7	4.0, 8.3	3.8, 9.0
**60 min**				
*Number with data (%)*	*21* (100)	*21* (95.5)	*22* (100)	20 (100)
Mean (SD)	6.4 (1.2)	6.8 (1.2)	6.3 (0.9)	6.4 (1.6)
Median (IQR)	6.6 (5.8, 7.0)	6.8 (6.1, 7.6)	6.6 (5.4, 7.1)	6.5 (5.3, 7.7)
Min, Max	3.8, 8.8	4.5, 9.6	4.4, 7.5	3.8, 8.9

### Harms

There were no adverse events while undertaking this study.

## Discussion

The primary outcome measure of this study aimed to quantitatively determine SARS-CoV-2 levels in saliva at various time points before and after mouthwash use. In this study viable virus titre was obtained from 42 out of 92 cases at baseline, due to the skewed distribution of the data, it was not possible to statistically analyse the primary outcome. Observationally the primary outcome data does show a reduction of virus titre using logarithm (base 10) of the focus forming units per millilitre at 1 min when all mouthwashes are compared to water.

Clinical trials in this field generally use PCR data as the primary outcome measure [13,17]. SARS-CoV-2 PCR testing has the advantage of being widely available and understood by the general population. A limitation of this technique is that it does not distinguish between viral RNA from viable, infectious SARS-CoV-2 virus and viral RNA from non-viable virus, so does not necessarily infer infectivity. Virus culture studies have shown that the ability to culture virus from infected patients reduces significantly from approximately a week post-symptom onset despite PCR positivity lasting 2 weeks or more [18]. Patient PCR positivity is therefore not a reliable indicator of whether an individual is able to shed infectious virus. In this study our objective was to quantitatively assess the effect of commercial mouthwashes on viable SARS-CoV-2 using virus culture, in addition to PCR positivity.

Pickering *et al.* [19] demonstrated that not all SARS-CoV-2 PCR positive patients gave a positive result in virus culture tests. In this study 57 out of 141 patients were both PCR and culture positive which is in line with our results. To improve the chances of participants being both swab and culture positive we asked participants to confirm LFT positivity on the day of sampling. We focussed on community-based infection during recruitment as it was felt the hospital inpatients had been infected longer and would have a reduced chance of being both swab and culture positive.

We have demonstrated and it is widely accepted that SARS-CoV-2 virus is found in saliva[20, 21]. On average saliva flow is 0.3–0.4ml min^−1^ [22]. It is reasonable to deduce that the mouthwashes initial effect on saliva SARS-CoV-2 levels is diminished as fresh saliva containing SARS-CoV-2 is produced soon after the mouthwashes use.

Multiple randomised controlled trials investigating mouthwashes effect on SARS-CoV-2 are available in the literature [23,26]. These studies generally use PCR data as the primary outcome measure. We aimed to quantitatively determine SARS-CoV-2 levels in saliva at various time points using viable virus titre, this is the point of differentiation of this study. We were unable to statistically analyse this outcome for reasons indicated. An outcome that predictably and accurately demonstrates the amount of viable virus in a saliva sample would be advantageous compared to PCR outcome data.

The mouthwashes included in this trial did observationally demonstrate a reduction in titre level 1 min after using all included mouthwashes, with virus levels increasing up to 60 min compared to the control. Although interesting it was not possible to statistically analyse this effect. Using the secondary outcome PCR measure all included mouthwashes reduced virus levels compared to water at 1 min and these results were statistically significant. This study shows that included mouthwashes reduce the amount of genetic material of SARS-CoV-2 in saliva 1 min after consumption of the mouthwash, but that this reduction is not substantive. Clinically this result does not support the use of the included mouthwashes to reduce SARS-CoV-2 levels in saliva. The results of this trial do not support the use of included mouthwashes prior to an aerosol generating oral procedure.

## Abbreviations

SARS-CoV-2: severe acute respiratory syndrome coronovirus 2
UKHSA: United Kingdom Health Security Agency’s
